# Percutaneous microwave ablation of hyperfunctioning parathyroid lesions under multiparametric ultrasound guidance for primary hyperparathyroidism: a systematic review and meta-analysis

**DOI:** 10.3389/fonc.2026.1921204

**Published:** 2026-09-21

**Authors:** Mei Kei Lum, Jie Wang, Zibo Ye, Bolin Wu, Fedor Petrovich Vetshev, Natalia Nikolaevna Vetsheva, Jung Hwan Baek, Maksim Boshrovish Saliba

**Affiliations:** 1 Department of Hospital Surgery No. 1, N.V. Sklifosovsky Institute of Clinical Medicine, I.M. Sechenov First Moscow State Medical University (Sechenov University), Ministry of Health of the Russian Federation, Moscow, Russia; 2 Department of Ultrasound, Harbin Medical University Cancer Hospital, Harbin, China; 3 Department of Radiology and Research Institute of Radiology, University of Ulsan College of Medicine, Asan Medical Center, Songpa-Gu, Seoul, Republic of Korea

**Keywords:** biochemical response, complication, meta-analysis, microwave ablation, multiparametric ultrasound, primary hyperparathyroidism

## Abstract

**Background:**

Multiparametric ultrasound (MPUS)-guided percutaneous microwave ablation (MWA) has emerged as a minimally invasive treatment option for primary hyperparathyroidism (PHPT), but pooled evidence on longitudinal biochemical outcomes and safety remains limited.

**Objective:**

This meta-analysis aimed to evaluate the efficacy and safety of percutaneous MWA performed under ultrasound guidance for PHPT.

**Methods:**

Following PRISMA guidelines, eligible cohort studies were retrieved from five electronic databases. Pooled cure rates, serial changes in intact parathyroid hormone (iPTH), serum calcium and phosphorus, overall complication rates, and adenoma volume reduction were calculated via random-effects models. Leave-one-out sensitivity analyses were performed to assess the robustness of the pooled estimates.

**Results:**

Nine cohorts enrolling 355 PHPT patients were included. Technical success was 100%. Pooled clinical cure rates reached 86.0% at 6 months, 82.0% at 12 months, and 75.0% at the last available follow-up. Serum calcium and iPTH declined significantly, and serum phosphorus rose across all time points. The pooled patient-level overall complication rate was 11.0%, with transient hoarseness and transient hypocalcemia being the most commonly reported event-specific complications, whereas permanent recurrent laryngeal nerve injury was rare.

**Conclusion:**

Current evidence suggests that percutaneous MWA performed under ultrasound guidance may be a minimally invasive treatment option for appropriately selected patients with PHPT, providing acceptable mid-term biochemical improvement with a tolerable safety profile.

**Systematic Review Registration:**

https://www.crd.york.ac.uk/PROSPERO/view/CRD42025644973, identifier CRD42025644973

## 1 Introduction

Primary hyperparathyroidism (PHPT) is a common endocrine disorder characterized by autonomous over-secretion of parathyroid hormone (PTH). Excess PTH leads to hypercalcemia, accelerated bone resorption, and a series of systemic metabolic complications (1, 2). For decades, open neck exploration combined with targeted parathyroidectomy has served as the mainstream treatment approach, achieving biochemical cure rates above 95% in routine clinical practice (3). Nevertheless, conventional open surgery carries inherent risks. These include complications related to general anesthesia, permanent cervical scarring, and iatrogenic damage to adjacent vital structures, among which recurrent laryngeal nerve palsy and persistent hypoparathyroidism are the most clinically concerning (4, 5). Reoperative surgery carries substantially higher complication risks than primary surgery (6, 7).

In view of the morbidity associated with repeat surgery, clinicians have increasingly turned to thermal ablation, which uniquely allows for tissue conservation and future retreatments. As for therapeutic outcomes, extant studies substantiate that microwave ablation (MWA) offers consistent and effective management of PHPT (8). Radiofrequency ablation (RFA) was introduced earlier for PHPT (9), and MWA has subsequently gained attention for its biophysical advantages (10). Previous pooled analyses have validated the general feasibility of multiple thermal modalities, including radiofrequency and laser ablation, for this disorder (11). Conventional B-mode ultrasound was the sole guidance tool in most early ablation procedures. Due to poorly defined lesion margins, operators are often required to reposition ablation needles repeatedly. Repeated repositioning of the ablation probe not only prolongs operation time but also raises the risk of incomplete ablation (12). Unlike other thermal ablation modalities, MWA has unique biophysical characteristics. Its energy transmission is minimally affected by tissue conductivity, supporting rapid heating, while limiting tissue carbonization and reducing heat-sink effects (13).

As a key component of the multiparametric ultrasound (MPUS) protocols, intraoperative contrast-enhanced ultrasound (CEUS) allows clinicians to visualize feeding vessels and tissue perfusion within target lesions (14). Clinicians can adopt more refined ablation tactics alongside standard workflows: moving-shot and multipoint ablation performed with MPUS guidance. Such improved operating steps simplify antenna positioning and noticeably cut down overall procedural time (15). In current clinical practice, hydrodissection has become a standard auxiliary step during MWA procedures. By injecting fluid around the recurrent laryngeal nerve, this technique builds a physical and thermal barrier to protect the nerve from thermal damage while high thermal energy is delivered to target lesions (16).

Several systematic reviews and meta-analyses have examined percutaneous thermal ablation for hyperparathyroidism in recent years (8, 11, 17, 18). Most combined different ablation modalities, enrolled patients with different forms of hyperparathyroidism, or did not distinguish ultrasound guidance protocols. This review differs by focusing exclusively on MWA for PHPT while incorporating the latest available evidence and providing a detailed assessment of procedural characteristics, efficacy, safety, and long-term outcomes. A comparison with previous reviews is presented in **Table 1**.

**Table 1:** Comparison of the present review with previous systematic reviews and meta-analyses of thermal ablation for PHPT.

Review (year)	PHPT only	MWA only	US guidance evaluated	Search	studies (patients)	Major outcomes	Key distinction
Ye/2020 (11)	Yes	No (MWA+RFA)	No	Oct 2019	5 (84)	Biochemical outcomes, gland volume reduction, and safety	Combined different thermal ablation modalities; US guidance protocols were not specifically evaluated.
Xu/2023 (17)	No (PHPT+SHPT)	No (MWA vs. RFA)	No	Dec 2022	5 (488)	Biochemical response, procedural time, complications	Compared MWA and RFA rather than differences in US guidance protocols.
Bilgin/2025 (8)	Yes	No (MWA+RFA+ethanol ablation)	No	Jan 2024	20 (815)	Normocalcaemia, treatment success, complications	Included multiple percutaneous ablation modalities; US guidance protocols were not systematically evaluated.
Jeong/2025 (18)	No (PHPT+SHPT+THPT)	No (RFA only)	No	Jul 2024	14 (593)	Biochemical outcomes, volume reduction, safety	Restricted to RFA and incorporated multiple subtypes of hyperparathyroidism.
Present study	Yes	Yes	Yes*	Apr 2026	9 (355)	Clinical cure, biochemical outcomes, VRR, safety	Restricted to MWA for PHPT; incorporates updated evidence; summarizes heterogeneous US guidance protocols; and comprehensively evaluates procedural characteristics, efficacy, safety, and long-term outcomes.

PHPT, primary hyperparathyroidism; SHPT, secondary hyperparathyroidism; THPT, tertiary hyperparathyroidism; MWA, microwave ablation; RFA, radiofrequency ablation; VRR, volume reduction rate; CEUS, contrast-enhanced ultrasound; US, ultrasound.

*US guidance protocols were descriptively categorized according to the imaging modalities used (B-mode ultrasound plus color Doppler imaging only versus additional CEUS). Formal subgroup meta-analysis was not performed because only two studies did not use CEUS.

As the clinical application of percutaneous MWA for PHPT continues to expand, questions remain regarding its long-term efficacy, safety, and technical implementation. Published clinical cohorts have yielded conflicting results regarding longitudinal changes in intact PTH (iPTH) levels, complication profiles, and long-term treatment efficacy (19, 20) Therefore, we present a systematic review and meta-analysis to synthesize the available evidence, evaluating technical success, clinical cure, serial biochemical responses, lesion volume reduction, procedural safety, and ultrasound guidance characteristics.

## 2 Materials and methods

This meta-analysis was performed in line with the PRISMA reporting guidelines (21). The study protocol was prospectively registered on PROSPERO (Registration No. CRD42025644973).

### 2.1 Literature search strategy

A systematic literature search was carried out on April 1, 2026, across five electronic databases: PubMed, EBSCOhost (Academic Search Ultimate), Cochrane Library, Scopus, and Web of Science. The search terms included microwave ablation, multiparametric ultrasound, and parathyroid disorders, as detailed in **Supplementary Table S1**. MPUS guidance was defined according to the terminology proposed by Sidhu et al. as the use of two or more complementary ultrasound modalities. Across the included studies, MPUS comprised different combinations of B-mode ultrasound, color Doppler imaging, CEUS, and ultrasound elastography rather than a standardized imaging protocol (22, 23).

We constructed an extensive preliminary search plan to boost retrieval sensitivity and avoid missing any papers matching our preset inclusion standards. Subsequently, strict eligibility screening was performed. Only original studies focusing on multiparametric ultrasound-guided microwave ablation for primary hyperparathyroidism were retained to guarantee the homogeneity and credibility of the subsequent pooled analysis.

### 2.2 Inclusion and exclusion criteria (PICOS framework)

According to the PICOS principle, the inclusion criteria were defined as follows:

Population (P): patients with a confirmed diagnosis of primary hyperparathyroidism, regardless of age, sex, or ethnicity.

Intervention (I): microwave ablation performed under multiparametric ultrasound guidance, defined *a priori* as a combined ultrasound approach integrating at least two of the following modalities: B-mode ultrasound, color Doppler imaging, contrast-enhanced ultrasound (CEUS), and ultrasound elastography. This definition adheres to established standards reported in prior studies (20, 21).

Comparison (C): not applicable for the single-arm cohorts that constituted the primary pooled analysis. Comparative data from studies with surgical or other ablation comparators were described narratively.

Outcomes (O): biochemical cure rates, serial changes in serum intact parathyroid hormone (iPTH), serum calcium, serum phosphorus, adenoma volume reduction, and procedural complications. A minimum follow-up of 6 months was required for medium-term outcome evaluation.

Study design (S): prospective or retrospective clinical cohort studies published as full-text original articles in English.

The studies were excluded based on the following criteria: (1) Non-original literature, such as reviews, conference abstracts, letters, and case reports; (2) Studies focusing on secondary or tertiary hyperparathyroidism; (3) Combined thermal ablation cohorts without separable pure MWA data; (4) Ablation procedures guided by single-mode rather than multiparametric ultrasound; (5) Studies with overlapping or duplicate patient cohorts from the same medical center; (6) *In vitro*, cell line, or animal experimental studies.

### 2.3 Data extraction and quality assessment

Two independent reviewers extracted data using a standardized data collection form, which included study design, baseline characteristics, ablation parameters, follow-up intervals, serum iPTH, calcium, phosphorus levels, and complications. The included studies reported serum calcium measurements; however, they did not consistently specify whether these represented total, albumin-corrected, or ionized calcium. Similarly, information regarding iPTH assay platforms and reference ranges was not consistently reported across the included studies. Outcome evaluations were conducted at 1 week, 1 month, 6 months,12 months after ablation and the final follow-up. Furthermore, clinical cure was defined as the concurrent normalization of serum calcium and iPTH at the corresponding follow-up time points. Only outcome data meeting both biochemical criteria were included in the pooled analyses of clinical cure. Pooled analyses were performed separately for each predefined follow-up interval using all eligible outcome data available at the corresponding follow-up time point. We included only pure microwave ablation studies and merged overlapping reports from the same cohort to avoid duplicate patient inclusion. When multiple publications reported overlapping patient cohorts, outcome data were extracted from the publication providing the most complete and relevant information for each analysis while avoiding duplicate patient inclusion. Details of the data extraction strategy for overlapping patient cohorts are provided in **Supplementary Table S2**.

Study quality was assessed using the Newcastle–Ottawa Scale (NOS) (24). The standard 9-star NOS was applied for comparative studies, while a modified 7-star version excluding the comparability domain was used for single-arm cohorts (25). The scale evaluated cohort representativeness, recruitment, baseline PHPT status, objectivity of measurements, follow-up adequacy and participant attrition. We defined high quality as ≥7 stars for comparative studies and ≥5 stars for single-arm studies. Any disagreements during data extraction and quality assessment were settled by consensus or a third reviewer.

### 2.4 Definition of complications

Complications were classified in accordance with the Society of Interventional Radiology (SIR) and Korean Society of Thyroid Radiology guidelines (KTA) (26–28). Major complications were defined as life-threatening events, those requiring invasive intervention, or permanent functional deficits persisting more than 12 months, including permanent recurrent laryngeal nerve palsy or permanent hypoparathyroidism. Minor complications were transient adverse events resolving within 12 months (SIR Grade B), subdivided into procedural injuries (transient hoarseness, localized hematoma) and biochemical events (transient hypoparathyroidism or hypocalcemia, requiring temporary supplementation). Side effects (e.g., mild pain, fever, asymptomatic fluctuations) were excluded from pooled analyses. Patient-level overall complications were analyzed only in studies where the number of patients experiencing at least one complication could be reliably reconstructed from the original reports. Studies without reconstructable patient-level data were excluded from the pooled overall complication analysis but remained eligible for event-specific analyses.

### 2.5 Statistical analysis

All analyses were performed in R (version 4.5.0) using the *meta* package. Single-arm dichotomous outcomes (technical success, clinical cure, and complication rates) were pooled using a random-effects DerSimonian-Laird model (29), with logit transformation to stabilize variance for bounded event rates (30). Continuous parameters, including iPTH, serum calcium, and serum phosphorus, were reported as mean differences (MDs) with 95% confidence intervals (CIs) to reflect post-ablation changes from baseline. All MD values were calculated as pre-ablation baseline minus post-ablation follow-up values. A positive MD indicates a postoperative reduction, while a negative MD indicates a postoperative increase.

Random-effects models were used throughout to account for cross-center clinical and methodological variability. Data were presented descriptively when extreme heterogeneity prevented quantitative pooling (31). Heterogeneity was assessed using the I² statistic and Cochran’s Q test, and a leave-one-out sensitivity analysis was conducted to test the stability of pooled results (**Supplementary Figure S1**). As a sensitivity analysis, the major pooled outcomes were reanalyzed using restricted maximum likelihood (REML) estimation with Hartung–Knapp adjusted confidence intervals and 95% prediction intervals to evaluate the robustness of the primary random-effects analyses (**Supplementary Table S3**).

Study-level weighted meta-regression was applied to examine the association between maximum adenoma diameter and total ablation energy (32). In line with Cochrane Handbook recommendations, publication bias was not assessed, given fewer than 10 studies in each analysis (31). All tests were two-sided, and P < 0.05 was defined as statistically significant.

## 3 Results

### 3.1 Literature search and study characteristics

A total of 560 articles were retrieved from five electronic databases. After removing 134 duplicates, 426 records underwent title and abstract screening, and 402 irrelevant entries were excluded according to preset inclusion criteria. Notably, two pairs of publications were reported by Liu et al. (33, 34). and Wei et al. (35, 36) were identified as overlapping publications describing the same patient cohorts with complementary outcome data. To avoid duplicate patient inclusion while maximizing data completeness, data from these overlapping publications were integrated according to the predefined data extraction strategy. Details of the data extraction process are provided in **Supplementary Table S2**.Finally, 9 independent clinical cohorts derived from 11 eligible reports were included for quantitative analysis (**Figure 1**).

**Figure 1:**
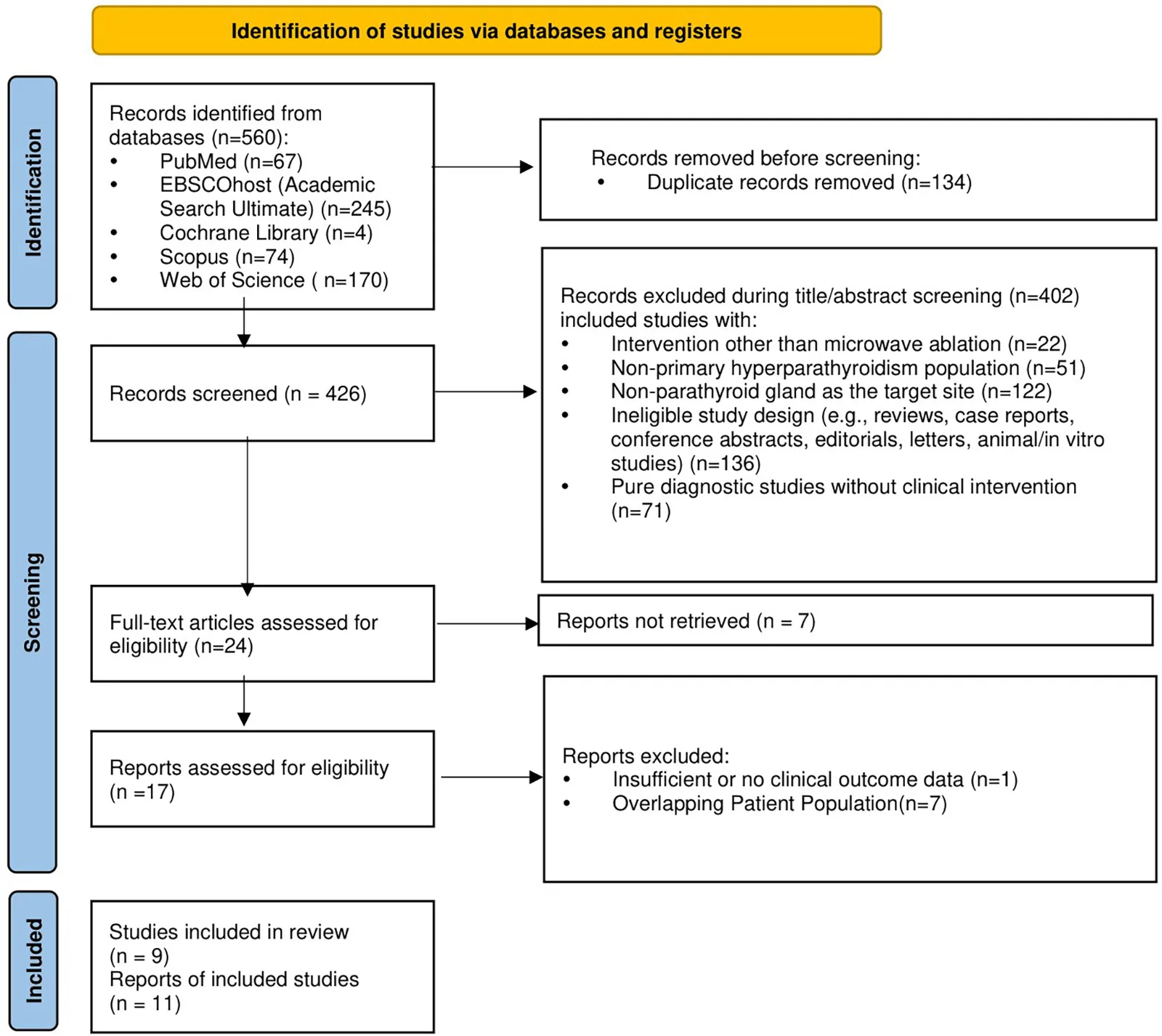
PRISMA flow diagram for study selection. A total of 11 articles were assessed; 9 independent cohorts were included following removal of overlapping patient data.

The total sample size was 355 patients, with all included non-randomized trials published between 2016 and 2026. The mean participant age ranged from 45.45 to 60.04 years. Preoperative maximum adenoma diameter varied from a mean of 1.09 cm to a median of 1.87 cm. Baseline iPTH concentrations (122.35–592.5 pg/mL) and serum calcium levels (2.68–2.93 mmol/L) confirmed biochemically active PHPT before intervention. Post-ablation follow-up durations ranged from 6 months to a mean of 32.8 months (**Table 2**).

**Table 2:** Baseline characteristics of the clinical cohorts included in the meta-analysis.

Author/ year	Study design	Study period	No.of patients	Patients (female/male)	Mean age(Y)	Max adenoma diameter (cm)	No. of lesions	Preoperative		Follow-up, months
								iPTH (pg/mL)	Calcium (mmol/L)	
Liu/2016 (37)	Retrospective	2011-2014	15	(9/6)	55.6 ± 14	2.28 ± 0.87	17	592.5 ± 579.1	2.93 ± 0.47	32.8 ± 17.9
Fan/2019 (38)	Retrospective	2014-2016	22	(19/3)	55.95 ± 12.95	1.40 ± 0.78	22	251.78 ± 249.42	2.72± 0.43	12.55 ± 5.21
Wu/2020 (39)	Retrospective	2016-2018	20	(12/8)	51.2 ± 13.5	NR	20	146.84 ± 103.68	2.78 ± 0.19	24
Guan/2026 (40)	Retrospective	2020-2024	25	(15/10)	60.04 ± 17.38	1.70 ± 0.63	25	322.15 ± 334.65	2.78 ± 0.37	7.48 ± 4.8
Zhou/2022 (41)	Retrospective	2018-2020	40	(28/12)	54.9 ± 11.8	1.09± 0.28	44	178.5 (134.5–229.3)	2.68 ± 0.24	11.6 m
Liu/2022* (33, 34)	Prospective Multicenter	2017-2020	91*	(59/32) *	56.80± 12.34	1.60 (1.20, 2.10)	84	137.30 (100.35, 202.40)	2.70 ± 0.28	12 (6-36)
Zhang/2022 (42)	Retrospective	2019-2021	35	(26/9)	55.63 ± 15.74	1.62 ± 0.64	38	171.31 ± 85.91	2.69 ± 0.11	6
Wei/2022** (35, 36)	Retrospective (PSM)	2015-2020	87	(57/30)	55.1 ± 16.8	1.40 (0.6–3.7)	97	201.2 (111.3–610.0)	2.8 ± 0.3	28.5 m
Ni/2021 (43)	Retrospective	2019-2021	20	(17/3)	45.45 ± 13.68	1.87 (1.45, 2.50)	21	122.35 (91.52, 266.90)	2.72 ± 0.30	6 m

Data are presented as mean ± standard deviation, median (range), or n (%). Abbreviations: iPTH, intact parathyroid hormone; PSM, propensity score matching; NR, not reported; m, median. All included studies were conducted in China.

*Liu et al. cohort: Data were pooled from multiple publications to avoid duplicate counting. The total sample size (n=91), gender distribution, and primary clinical cure rate (80.22%) were derived from the prospective multicenter trial (Liu 2022a). Baseline biochemical parameters, complication rates, and serial follow-up data were supplemented from the companion report (Liu 2022b, n=75).

**Wei et al. cohort: Data were integrated from two publications describing the same patient cohort. Baseline characteristics and primary outcomes were extracted from the propensity score-matched study (Wei 2022, n=87). Serial biochemical data across multiple follow-up time points were supplemented from the detailed follow-up report (Wei 2020, n=67).

### 3.2 Quality assessment and risk of bias

The methodological quality of the included studies is summarized in **Table 3**. Two comparative trials achieved Newcastle-Ottawa Scale scores of 8/9 and 9/9, both indicating high quality. The remaining seven single-arm studies had adjusted scores ranging from 5/7 to 6/7. Overall, three single-arm datasets were rated high quality, and four were rated moderate quality, supporting reliable quantitative synthesis.

**Table 3:** Methodological quality assessment of included studies.

Study ID	Study design & comparison	Selection	Comparability	Outcome	Total score	Quality tier
Comparative studies						
Liu/2022* (33, 34)	Prospective, Multicenter (MWA vs. RFA)	★★★★	★	★★	8/9	High Quality
Wei/2022** (35, 36)	Retrospective, PSM-matched (MWA vs. Surgery)	★★★★	★★	★★★	9/9	High Quality
Single-arm studies						
Liu/2016 (37)	MWA Only	★★★	N/A	★★★	6/7	High Quality (adjusted)
Fan/2019 (38)	MWA Only	★★★	N/A	★★★	6/7	High Quality (adjusted)
Wu/2020 (39)	MWA Only	★★★	N/A	★★	5/7	Moderate Quality
Guan/2026 (40)	MWA Only	★★★	N/A	★★	5/7	Moderate Quality
Zhou/2022 (41)	MWA Only	★★★	N/A	★★	5/7	Moderate Quality
Zhang/2022 (42)	MWA Only	★★★	N/A	★★★	6/7	High Quality (adjusted)
Ni/2021 (43)	MWA Only	★★★	N/A	★★	5/7	Moderate Quality

MWA, microwave ablation; N/A, not applicable; PSM, propensity score matching; RFA, radiofrequency ablation. The symbols ★, ★★, ★★★, and ★★★★ represent points awarded according to the Newcastle–Ottawa Scale (NOS) for methodological quality assessment. Each ★ corresponds to one point within the relevant NOS domain. Accordingly, ★ = 1 point, ★★ = 2 points, ★★★ = 3 points, and ★★★★ = 4 points. The stars are assigned across the selection, comparability, and outcome domains, and their sum corresponds to the total quality score reported for each study.

### 3.3 Ablation procedures and technical characteristics

All studies used B-mode imaging to assess parathyroid lesions prior to intervention, which were identified as solid nodules with either regular or irregular borders. Intraoperative assessment of perilesional blood flow was performed with CDUS; Liu et al. (2016) (37) and Wu et al. (2020) (39) utilized this tool to track vascular pedicles and steer the ablation applicator away from major vessels. The ultrasound guidance protocols differed across the included studies. Specifically, 7 studies used B-mode ultrasound, color Doppler imaging, and CEUS, whereas 2 studies [Fan et al., 2019 (38) and Guan et al., 2026 (40)] used B-mode ultrasound and color Doppler imaging only (**Table 4**). None of the included studies reported the use of elastography for procedural guidance. Intraprocedural hyperechoic signals generated by microbubbles enabled real-time monitoring throughout treatment. On immediate post-ablation CEUS, a1–2 mm peri-lesional non-enhancing rim on immediate post-ablation CEUS has been proposed as a potential imaging marker of complete ablation; however, this observation requires further clinical validation before it can be considered a definitive criterion.

**Table 4:** Technical parameters of microwave ablation in the included studies.

Study	US modality	MWA device	Applicator	Power (W)	Ablation time (s)	Total energy (kJ)	Ablation technique	Hydrodissection
Liu/2016	B-mode, CDUS, CEUS	KY-2000, Kangyou Medical	16G, 3/5-mm active tip	40	NR	NR	Moving-shot; vascular pedicle approach	NS
Fan/2019	B-mode, CDUS	KY-2000, Kangyou Medical	17G, 3-mm active tip	30–45	129.14 ± 122.55	4.84 (3.87–5.81)	Segmented (large lesion); fixed-point (small lesion)	4 °C cold saline injection (CCSI)
Wu/2020	B-mode, CDUS, CEUS	ECO-100 (YIGAO)	16G, 3-mm active tip	35	122.29 ± 107.54	4.28 (0.52–8.04)	Moving-shot; vascular pedicle approach	Lidocaine-mixed saline
Guan/2026	B-mode, CDUS	Greatwall, Nanjing	NR	20–30	NR	NR	Fixed-point	NS
Zhou/2022	B-mode, CDUS, CEUS	Nanjing ECO Microwave System	17G, 4-mm active tip	40–45	218.4 ± 37.9	9.28 (8.74–9.83)	Multipoint (large lesion); fixed-point (small lesion)	NS
Liu/2022*	B-mode, CDUS, CEUS	KY-2000, Kangyou Medical	16G/17G, 3-mm active tip	20–30	120 ± 45	3.00 (2.40–3.60)	Moving-shot (>10 mm); fixed-point (<10 mm)	NS
Zhang/2022	B-mode, CDUS, CEUS	Nanjing ECO Microwave System	16G	25–30	182.63 ± 101.06	5.02 (4.57–5.48)	Moving-shot	NS
Wei/2022**	B-mode, CDUS, CEUS	KY-2000, Kangyou Medical	17G, 3-mm active tip	30	170 (89–950)	5.10 (2.67–28.50)	Multipoint	NS
Ni/2021	B-mode, CDUS, CEUS	KY-2000, Kangyou Medical	16G, 3-mm active tip	30	72.00 (55–120)	2.16 (1.65–3.60)	Moving-shot (large); fixed-point (small)	NS

CCSI, continuous cool saline injection; NS, normal saline; NR, not reported.

Total ablation energy (kJ) = Power (W) × Ablation time (s)/1000. Mean power was used for calculation when a power range was presented. Data from overlapping Liu et al. publications were combined to prevent duplicate counting; parameters were sourced from Liu et al. (2022*). Data of Wei et al. (2020/2022) were synthesized from related studies, with technical details obtained from the 2020 prospective report. CEUS was used for real-time monitoring and treatment evaluation. Most studies adopted SonoVue (Bracco, Italy), and Zhou et al. (2022) used Sonazoid (GE Healthcare, Norway).

### 3.4 Technical success rate and clinical cure rates

All nine cohorts achieved a 100% technical success rate. Targeted ablation was successfully performed for 388 parathyroid lesions in 355 patients, with no intraoperative failures.

The 6-month cure rate from 3 cohorts was 86.0% (95% CI: 57.0%–97.0%; *I²* = 72.8%, *P* = 0.025) (**Figure 2A**). The 12-month cure rate from 4 cohorts was 82.0% (95% CI: 74.0%–88.0%; *I²* = 0.0%, *P* = 0.912) (**Figure 2B**). The final follow-up cure rate from 5 cohorts, was 75.0% (95% CI: 48.0%–91.0%; *I²* = 86.9%, *P* < 0.001) (**Figure 2C**).

**Figure 2:**
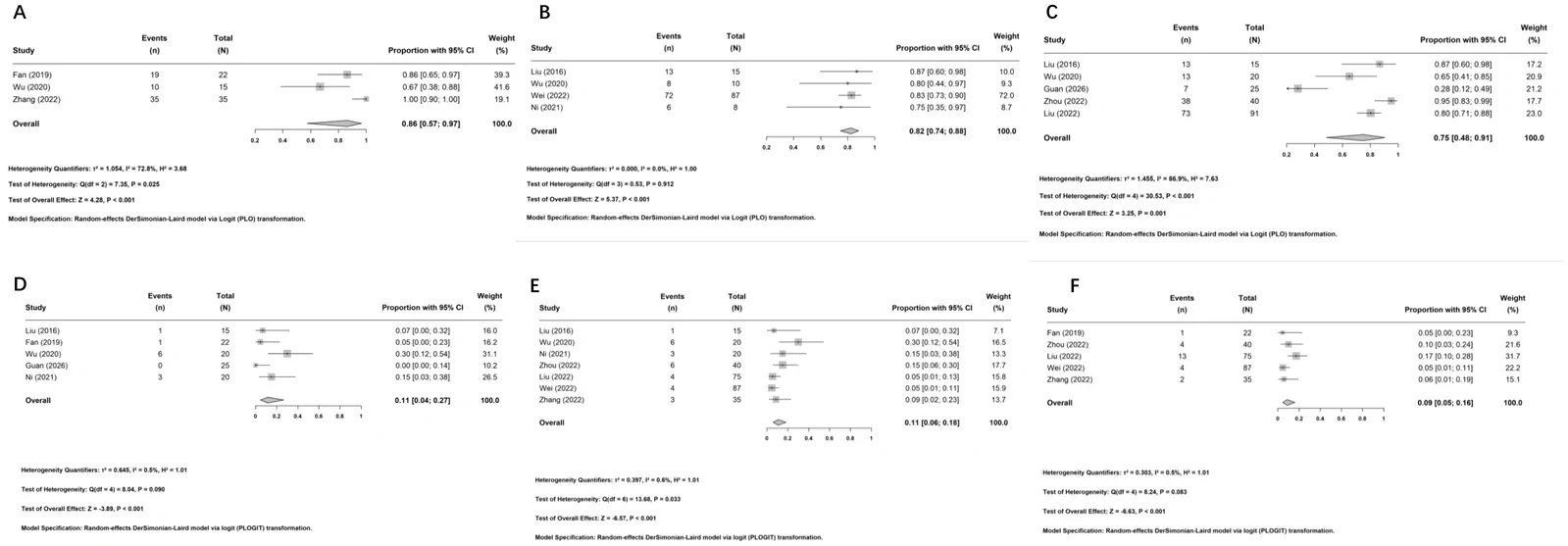
Forest plots of clinical outcomes and complications after ultrasound-guided MWA for PHPT. **(A–C)** Cure rates stratified by follow-up: **(A)** 6-month, **(B)** 12-month, **(C)** end-of-study. **(D)** Patient-level overall complication rate. **(E, F)** Minor complications: **(E)** transient hoarseness, **(F)** transient hypocalcemia. The pooled analysis in panel **(D)** included only studies in which patient-level overall complication rates could be reconstructed from the original reports. Random-effects models with logit transformation were used. CI, confidence interval; df, degrees of freedom; I², inconsistency index; MWA, microwave ablation; PHPT, primary hyperparathyroidism; Q, Cochran’s Q statistic.

### 3.5 Complication rates

The pooled patient-level overall complication rate based on five eligible studies was 11.5% (95% CI: 4.5%–26.6%; I² = 50.3%, P = 0.090) (**Figure 2D**). Major complications were extremely rare. Only Wei et al. (2022) (35) found two cases of permanent nerve paralysis leading to persistent hoarseness (2/87, 2.3%). The vast majority of complications were minor and self-limiting.

Separate pooled analyses were performed for transient hoarseness and transient hypocalcemia (**Figures 2E, F**). The pooled incidence of transient hoarseness was 11.0% (95% CI: 6.0%–18.0%) (**Figure 2E**), and most cases resolved spontaneously within 1–4 months. The pooled incidence of transient hypocalcemia was 9.0% (95% CI: 5.0%–16.0%) (**Figure 2F**). Detailed study-level adverse event data are provided in **Supplementary Table S4**.

#### 3.5.1 Intact parathyroid hormone (iPTH) levels

All included studies reported serum iPTH data (**Figure 3**). Significant iPTH reductions were observed at 1 week (4 studies; WMD = 108.06 pg/mL, 95% CI: 76.65–139.47, p < 0.001, I² = 91.6%), 1 month (6 studies; WMD = 90.42 pg/mL, 95% CI: 63.08–117.75, p < 0.001, I² = 92.6%), 6 months (8 studies; WMD = 119.18 pg/mL, 95% CI: 82.86–155.50, p < 0.001, I² = 94.8%), 12 months (6 studies; WMD = 100.03 pg/mL, 95% CI: 69.92–130.15, p < 0.001, I² = 95.8%) and final follow-up (5 studies; WMD = 147.46 pg/mL, 95% CI: 66.86–228.05, p = 0.003, I² = 74.9%). Subgroup analysis showed no temporal differences (p = 0.580), indicating sustained iPTH suppression.

**Figure 3:**
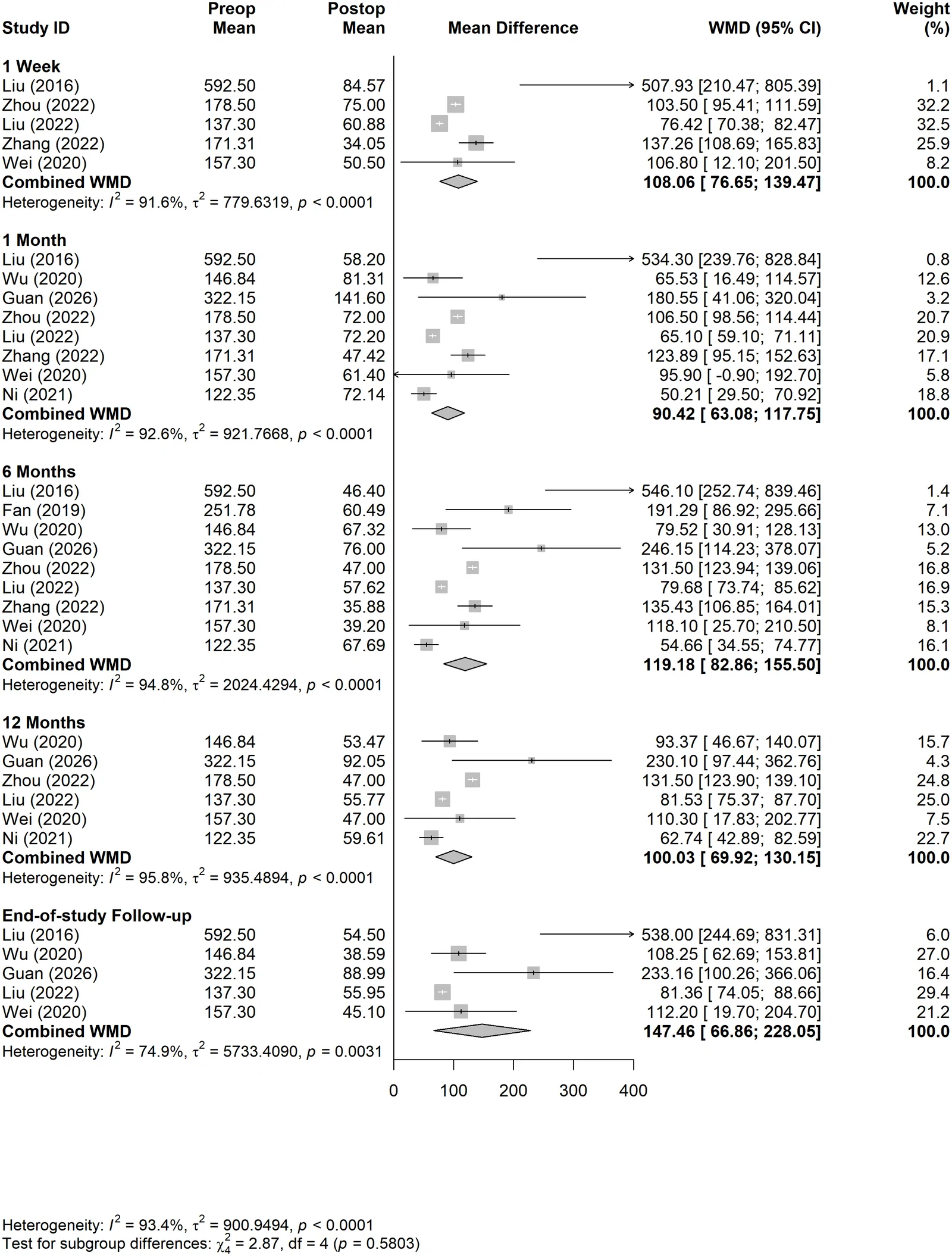
Forest plots showing iPTH levels in PHPT patients before and after MWA.

#### 3.5.2 Calcium levels

Serum calcium decreased significantly at 1-week post-ablation (**Figure 4**; WMD = 0.39 mmol/L, 95% CI: 0.34–0.45, *p* < 0.001, *I²* = 54.2%). The effect remained stable over time: 0.38 mmol/L at 1 month (95% CI: 0.35–0.41, *p* < 0.001, *I²* = 3.7%), 0.39 mmol/L at 6 months (95% CI: 0.35–0.43, *p* < 0.001, *I²* = 40.9%), 0.36 mmol/L at 12 months (95% CI: 0.32–0.40, *p* < 0.001, *I²* = 14.8%) and 0.36 mmol/L at final follow-up (95% CI: 0.31–0.42, *p* < 0.001, *I²* = 48.5%). No between-timepoint differences were found (*p* = 0.773), with calcium levels persistently lowered.

**Figure 4:**
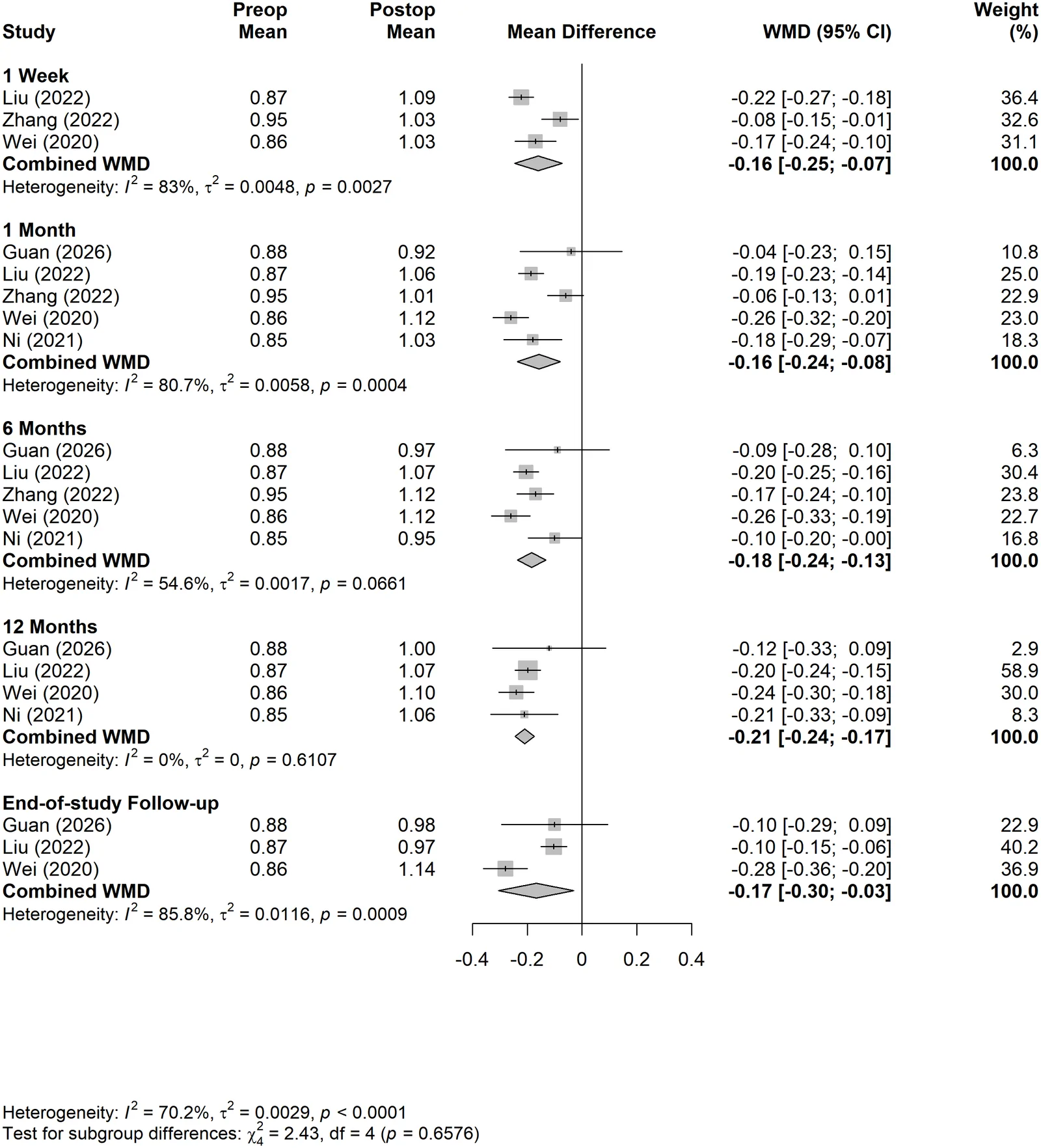
Forest plots showing calcium levels in PHPT patients before and after MWA.

#### 3.5.3 Phosphorus levels

Serum phosphorus increased after microwave ablation (**Figure 5**). At 1 week (3 studies; WMD = −0.16 mmol/L, 95% CI: −0.25 to −0.07, *p* = 0.003, *I²* = 83%), benefits persisted across follow-up: −0.16 mmol/L at 1 month (5 studies; 95% CI: −0.24 to −0.08, *p* < 0.001, *I²* = 80.7%), −0.18 mmol/L at 6 months (5 studies; 95% CI: −0.24 to −0.13, *p* < 0.001, *I²* = 54.6%), −0.21 mmol/L at 12 months (4 studies; 95% CI: −0.24 to −0.17, *p* < 0.001, *I²* = 0%) and −0.17 mmol/L at final follow-up (3 studies; 95% CI: −0.30 to −0.03, *p* = 0.009, *I²* = 85.8%). No rebound occurred, and treatment effects were consistent over time (*p* = 0.658).

**Figure 5:**
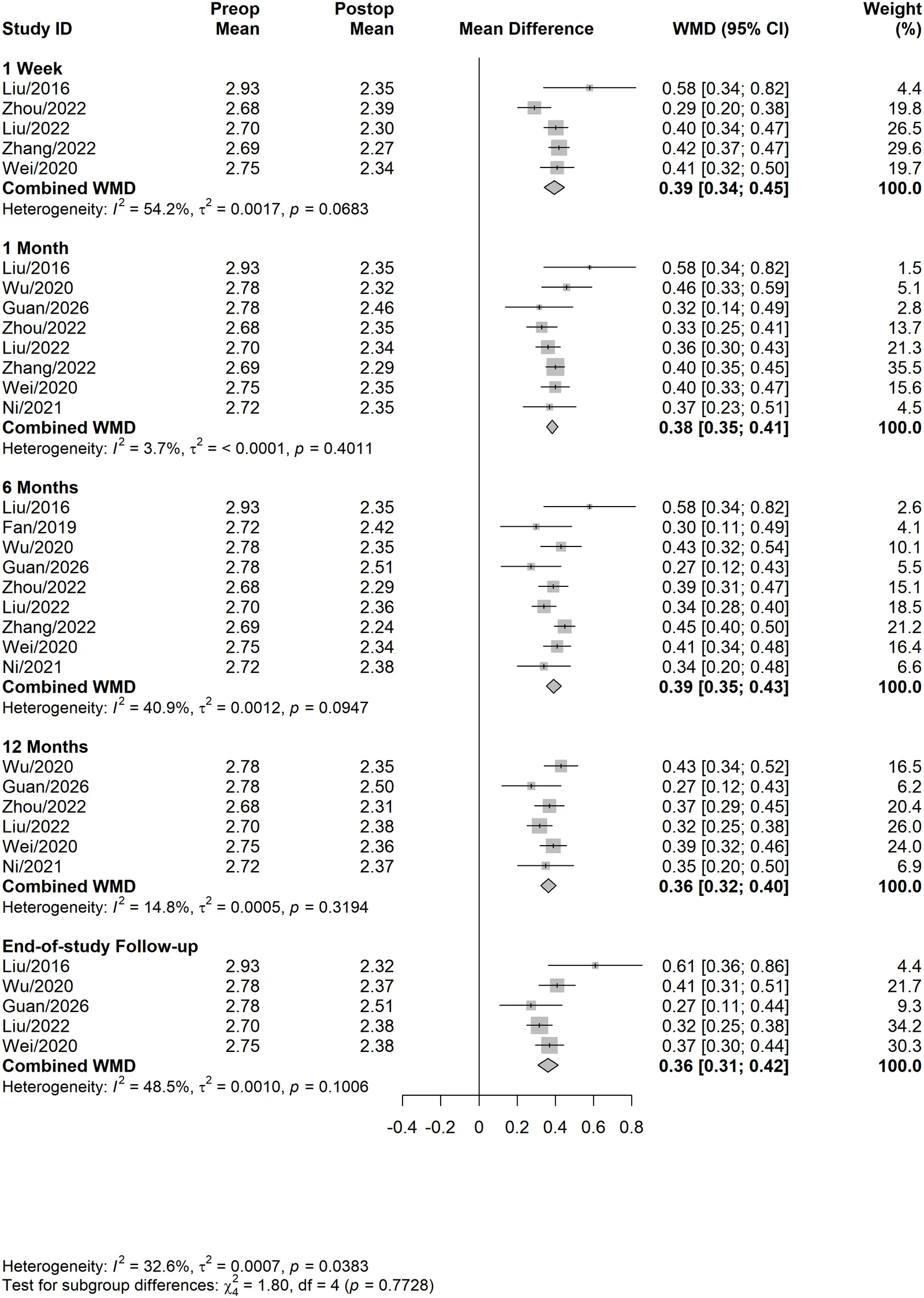
Forest plots showing phosphorus levels in PHPT patients before and after MWA.

### 3.6 Longitudinal changes in biochemical indices and lesion size

Pooled analyses were performed independently for each predefined follow-up interval using the eligible outcome data available at the corresponding follow-up time point. As shown in **Table 5**, biochemical and morphological improvements were sustained after ablation. Therapeutic effects on serum calcium and phosphorus remained stable throughout follow-up (*P* = 0.657 and 0.814, respectively), while reductions in iPTH showed mild temporal variation (*P* = 0.027). High inter-study heterogeneity was detected for iPTH, whereas other indicators had low-to-moderate heterogeneity. Parathyroid adenomas shrank progressively and significantly from 6 to 12 months post-operation. Study-level serum calcium and iPTH data at the last available follow-up are provided in **Supplementary Table S5**.

**Table 5:** Stratified longitudinal trajectories, pooled effect sizes, and stratified homogeneity profiles of continuous parameters post-MWA.

Outcome metric & follow-up group	No. of studies (k)	Total patients (N)	Pooled WMD (pre-post; 95% CI)	Effect size (Z)	P-value	Heterogeneity (I²; %)	Q-statistic (df)	Heterogeneity P
Serum calcium (mmol/L)								
1 Week	5	232	0.39 (0.34, 0.45)	13.7	<0.001	54.2	4.37 (4)	0.068
1 Month	8	297	0.38 (0.35, 0.41)	25.2	<0.001	3.7	7.27 (7)	0.401
6 Months	9	319	0.39 (0.35, 0.43)	18.62	<0.001	40.9	13.53 (8)	0.095
12 Months	6	247	0.35 (0.32, 0.40)	17.38	<0.001	10.6	4.69 (5)	0.319
End-of-study Follow-up	5	202	0.36 (0.31, 0.42)	13.09	<0.001	47	7.77 (4)	0.101
Serum intact PTH (pg/mL)								
1 Week	5	232	108.06 [76.65, 139.47]	6.35	<0.0001	91.6	47.64 (4)	<0.0001
1 Month	8	297	90.42 [63.08, 117.75]	6.46	<0.0001	92.6	94.68 (7)	<0.0001
6 Months	9	319	119.18 [82.86, 155.50]	6.43	<0.0001	94.8	153.97 (8)	<0.0001
12 Months	6	247	100.03 [69.92, 130.15]	6.5	<0.0001	95.8	94.58 (5)	<0.0001
End-of-study Follow-up	5	202	147.46 [66.86, 228.05]	3.58	0.0003	74.9	15.96 (4)	0.003
Serum phosphorus (mmol/L)								
1 Week	3	177	`-0.16 (-0.25, -0.07)	-3.49	<0.001	83	11.76 (2)	0.0027
1 Month	5	242	`-0.16 (-0.24, -0.08)	-3.92	<0.001	80.7	20.73 (4)	0.0004
6 Months	5	242	`-0.18 (-0.24, -0.13)	-6.41	<0.001	54.6	8.81 (4)	0.0661
12 Months	4	187	`-0.21 (-0.24, -0.17)	-11.73	<0.001	0	3.00 (3)	0.6107
End-of-study Follow-up	3	167	`-0.17 (-0.30, -0.02)	-2.38	0.0173	85.8	14.08 (2)	0.0009
Adenoma max diameter (cm)								
-- 6 Months	3	110	0.58 (0.31–0.85)	4.21	<0.001	22.1%	2.56 (2)	0.278
-- 12 Months	3	110	0.84 (0.54–1.14)	5.44	<0.001	11.5%	2.24 (2)	0.326

CI, confidence interval; df, degrees of freedom; k, number of cohorts; N, cumulative sample size; WMD, weighted mean difference. Reference ranges: calcium 2.1–2.6 mmol/L; iPTH 15–65 pg/mL; phosphorus 0.81–1.45 mmol/L. Subgroup difference: calcium P = 0.6574, iPTH P = 0.0273, phosphorus P = 0.8142.

### 3.7 Volume Reduction Rate (VRR)

At 6 months post-ablation, three studies reported VRR outcomes ranging from 44.20% to 88.93%. Pooled analysis was not conducted due to extreme inter-study heterogeneity (*I²* = 99.7%), and the data are presented descriptively (**Figure 6A**). At 12 months, four studies with 184 patients yielded a random-effects pooled VRR of 90.86% (95% CI: 83.56%–98.17%), with high heterogeneity (*I²* = 98.0%). For long-term follow-up (≥18 months), three studies including 107 patients showed a pooled VRR of 86.55% (95% CI: 70.53%–100.00%) and substantial heterogeneity (*I²* = 94.4%).

**Figure 6:**
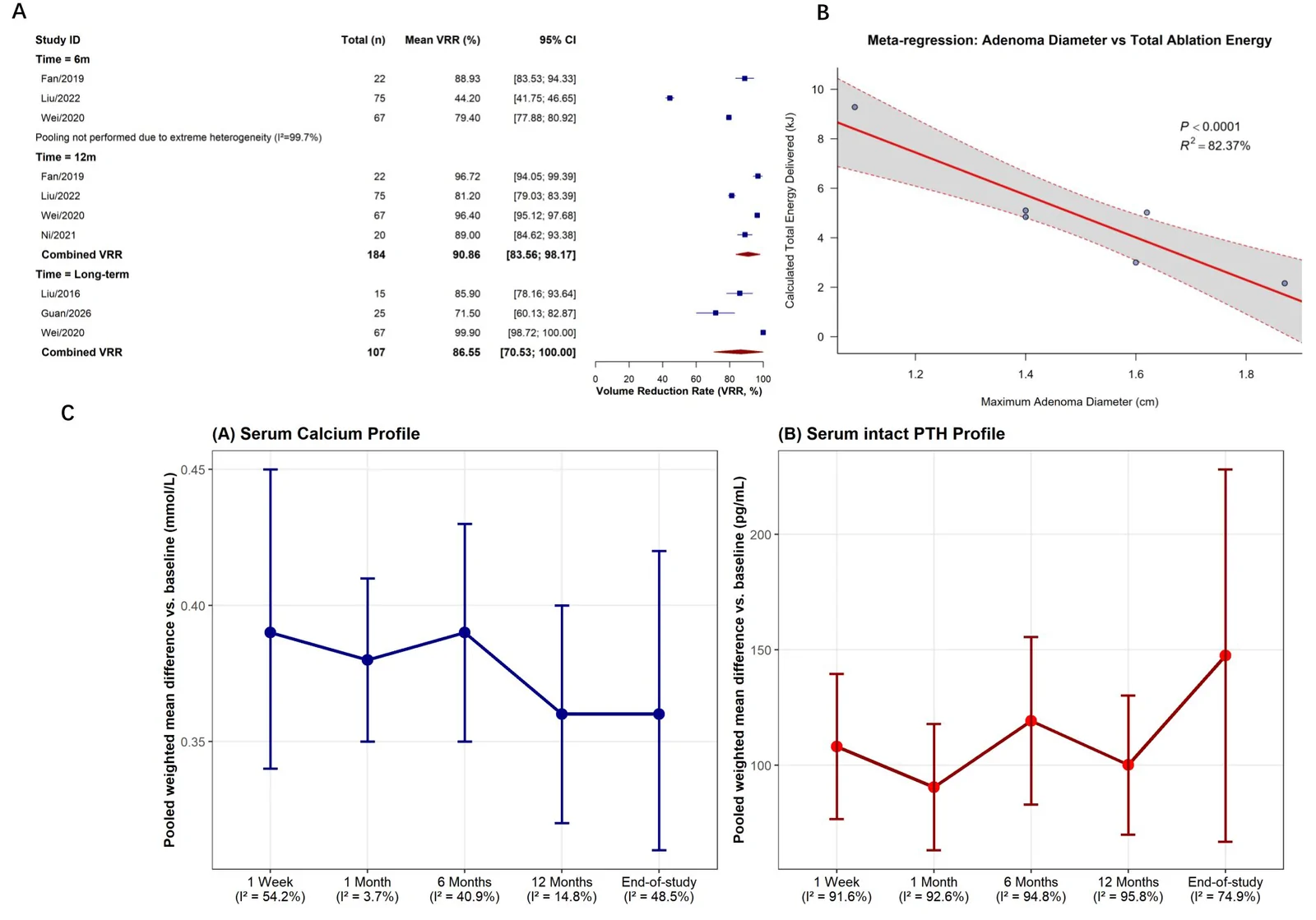
Treatment response and analysis of MWA for PHPT. **(A)** Forest plot of VRR by follow-up time. Pooling was omitted at 6 months due to extreme heterogeneity. **(B)** Meta-regression of adenoma diameter versus ablation energy. **(C)** Trajectory of WMDs for serum calcium and iPTH.

### 3.8 Exploratory study-level meta-regression analysis

An exploratory study-level meta-regression (**Figure 6B**) showed an inverse association between mean maximum adenoma diameter and total delivered ablation energy (*P* < 0.0001, *R²* = 82.37%). Given the limited number of studies, heterogeneous reporting of lesion size and ablation parameters, and the ecological nature of study-level meta-regression, this finding should be interpreted cautiously. It should not be interpreted as establishing a patient-level relationship. 

### 3.9 Longitudinal trajectories of serum calcium and iPTH responses

Serum calcium corrections were relatively stable over follow-up, with pooled reductions ranging from 0.36 to 0.39 mmol/L and narrow confidence intervals. The corresponding I^2^ values (3.7%–54.2%) indicated mild-to-moderate heterogeneity across time points. In contrast, iPTH reductions showed a more variable trajectory, with pooled decreases ranging from 89 to 147 pg/mL. High interstudy heterogeneity was observed across all iPTH subgroups (I2 74.9%–95.8%), reflecting greater between-study variability in parathyroid hormone responses (**Figure 6C**).

### 3.10 Sensitivity analyses

Leave-one-out sensitivity analyses were performed for the primary outcomes: 6-month clinical success rate (**Supplementary Figure S1A**), end-of-study clinical success rate (S1B), patient-level overall complication rate (S1C), and 12-month volume reduction rate (VRR; S1D). Excluding individual studies did not meaningfully alter pooled estimates for success rates or 12-month VRR, supporting the robustness of the primary findings. The favorable safety conclusion for microwave ablation remained unchanged. Sensitivity analyses using restricted maximum likelihood estimation, Hartung–Knapp adjusted confidence intervals, and 95% prediction intervals produced point estimates consistent with the primary DerSimonian–Laird analyses for all major outcomes. For outcomes with high heterogeneity and a limited number of included studies, confidence and prediction intervals were wider, reflecting greater statistical uncertainty in these outcomes (**Supplementary Table S3**).

## 4 Discussion

### 4.1 Summary of main findings

This systematic review and meta-analysis synthesized clinical evidence from nine independent cohorts to evaluate the efficacy and safety of MPUS-guided MWA for PHPT. Pooled estimates confirmed a 100% technical success rate, with mid-term clinical success rates of 86.0% at six months and 75.0% at the final follow-up (34, 37, 39–41), demonstrating its clinical value as an organ-preserving minimally invasive approach.

Zero heterogeneity at the 12-month follow-up is a statistical artifact attributable to limited sample size. Monitoring showed postoperative changes (36–39, 43): serum iPTH decreased significantly, serum calcium normalized, and serum phosphorus increased correspondingly, indicating the biochemical consequences of technical success.

### 4.2 Comparison with existing evidence

Earlier meta-analyses, such as the work reported by Ye et al. (11), pooled data from various thermal ablation techniques performed solely under conventional B-mode ultrasound. The present study has notable methodological refinements. In contrast, our analysis centers specifically on MPUS-assisted MWA. We adopted more selective inclusion rules and enrolled a larger volume of clinical cases, which helps generate more robust and all-round conclusions. All data processing and report preparation followed standard methodological guidelines to guarantee uniform study conduct (22). MPUS assists clinicians in pinpointing target lesions, mapping vascular structures and assessing tissue microperfusion. Intraoperative CEUS further displays vascular distribution and perfusion status in real time. This workflow helps mitigate heat-sink interference and produces evenly distributed coagulative necrosis. It has been suggested that a 1–2 mm non-enhancing band beyond the parathyroid gland boundary could serve as evidence of full ablation, yet relevant comparative evidence is still insufficient to fully validate this criterion (44).

Compared with conventional ultrasound, MPUS streamlines probe placement and cuts down total operative time (12). Replacing single-site ablation with moving-shot and multipoint strategies can further raise the rate of complete tissue ablation. The moving-shot ablation pattern enables complete circumferential coverage of targeted lesions. This technique eliminates residual hyperfunctioning parathyroid tissue and lower post-procedural recurrence risk. This conclusion is based on retrospective cohort data; therefore, prospective controlled studies are still required to verify the stability of this advantage.

Microwave ablation can trigger irreversible coagulative necrosis in overactive parathyroid cells. Over a median follow-up of 24–30 months, calcium and phosphorus levels stayed stable, whereas dynamic iPTH changes reflected physiological endocrine adaptation of parathyroid tissue. Hormonal suppression followed by gradual restoration of normal parathyroid function in the remaining normal parathyroid glands eventually leads to sustained biochemical stabilization (8, 45).

### 4.3 Clinical implications and patient selection

The 75.0% long-term success rate warrants objective interpretation. Failure to satisfy strict biochemical cure criteria does not equate to genuine treatment failure in all cases. Among the 25% of suboptimal cases, most exhibit normocalcemic iPTH elevation, defined as normal serum calcium alongside persistently elevated iPTH (46). Persistent normocalcemic iPTH elevation following MWA is likely multifactorial rather than attributable to a single mechanism. Potential contributing factors include vitamin D deficiency, inadequate calcium intake or supplementation, renal dysfunction, post-ablation bone remineralization (including hungry bone syndrome), residual hyperfunctioning parathyroid tissue, and unrecognized multiglandular disease (47, 48). Because individual patient-level data regarding these variables were not consistently reported across the included studies, the relative contribution of each mechanism could not be determined in the present review.

As summarized in **Table 2**, our cohort predominantly consisted of middle-aged and elderly women (mean age: 51–60 years; female proportion: ~68%). This demographic is susceptible to hungry bone syndrome following resolution of chronic hypercalcemia (48), a well-known cause of secondary iPTH elevation.

It is essential to distinguish technical success — complete target lesion ablation verified by MPUS— from clinical success, defined as normalization of both serum calcium and iPTH. Persistent biochemical abnormalities beyond six months may reflect several factors, including residual hyperfunctioning parathyroid tissue, unrecognized multiglandular disease, or post-treatment metabolic adaptation, rather than technical failure alone. Since MGD accounts for only 10–15% of sporadic PHPT cases (49), the remaining 85–90% of patients with carefully screened solitary adenomas are suitable for curative local ablation, achieving satisfactory efficacy and safety. Combined intraoperative MPUS effectively distinguishes fully ablated tissue from residual lesions, thereby facilitating long-term patient management and preventing unnecessary re-interventions (40, 41, 50).

Although parathyroidectomy achieves cure rates >95%, it carries risks of permanent hypoparathyroidism (approximately 1–2%), nerve injury, scarring, and anesthesia-related complications (5, 51–53). MPUS-guided MWA cannot replace open surgery. However, it offers a valuable organ-preserving alternative for three subgroups: (1) elderly patients with severe cardiopulmonary comorbidities; (2) patients who prioritize voice preservation and cosmetic outcomes and accept possible re-intervention; and (3) patients with a solitary, well-circumscribed parathyroid adenoma (preferably ≤1.5–2.0 cm) amenable to complete marginal ablation under MPUS guidance (54).

### 4.4 Technical considerations and operator dependence

The exploratory study-level meta-regression showed an inverse association between maximum adenoma diameter and total ablation energy (R² = 82.37%, P < 0.0001).

Because this meta-regression was based on a limited number of aggregate study-level observations with heterogeneous reporting of lesion size, ablation devices, power settings, antenna types, and partially indirectly calculated energy values, the observed association should be regarded as exploratory. The finding may reflect ecological or reporting artifacts and should not be interpreted as establishing a patient-level relationship. This exploratory analysis warrants confirmation in future studies with standardized reporting and individual patient-level data.

A key technical difference further influences operator behavior. RFA systems continuously monitor tissue impedance; a sharp rise triggers an automatic power shut-off, alerting the operator to potential thermal injury to surrounding structures (55). This safety feature is well recognized in clinical guidelines (27). In contrast, MWA generators lack this impedance-based feedback because microwave energy deposition is not constrained by tissue conductivity. Consequently, MWA is highly operator-dependent, with no automated safety alarm to prevent overtreatment near critical nerves.

### 4.5 Safety profile and heterogeneity sources

Leave-one-out sensitivity analyses confirmed robustness. Safety heterogeneity mainly originated from Wei et al. (2022) (35). Notably, this statistical discrepancy arose from the study’s strict postoperative follow-up scheme, rather than any technical flaws in its ablation procedures (19). Previous studies have suggested that transient hoarseness after MWA may compare favorably with other modalities and open surgery (56). The low rate of permanent nerve injury is closely related to routine hydrodissection, which forms a fluid barrier around the recurrent laryngeal nerve (57, 58). Because patient-level overall complication data were not reconstructable in several studies, the pooled overall complication analysis was restricted to studies with reconstructable patient-level data. Direct comparisons of complication rates with conventional parathyroidectomy or RFA were not feasible, as nearly all included studies were single-arm cohorts lacking comparator groups. Accordingly, the relative safety profile of MWA compared with these treatment modalities requires confirmation in future comparative studies.

### 4.6 Limitations

A key methodological limitation of the present review is that most included studies were single-arm observational cohorts without concurrent untreated control groups. Although pooled analyses demonstrated consistent biochemical improvement following MWA, causal inference remains limited as residual confounding and regression to the mean cannot be completely excluded. Future well-designed prospective controlled studies are needed to confirm these findings.

However, several limitations should be acknowledged. Differences in ablation hardware and center-specific procedural protocols among included study sites underpin the substantial heterogeneity in lesion volume reduction identified in our pooled analysis. Since every trial in this review was conducted in China, our conclusions cannot be readily applied to populations from other regions or ethnic origins. The pooled findings may also reflect local clinical practice, including operator expertise, patient selection, procedural protocols, and follow-up strategies. Therefore, independent validation in other populations is required before any conclusion regarding generalizability can be drawn. None of the included studies reported the use of elastography for procedural guidance, and reporting of microvascular imaging was inconsistent across studies. Additionally, ultrasound guidance protocols varied across the included studies, with two studies using B-mode ultrasound and color Doppler imaging without CEUS. Due to the limited number of non-CEUS studies (n=2), subgroup analyses according to imaging modality were not performed. Subgroup analyses or meta-regression based on baseline iPTH levels, adenoma location, and MWA procedural parameters were also not feasible because these variables were reported inconsistently or were unavailable across the included studies.

Moreover, long-term follow-up data insufficient to support more definitive conclusions. The clinical cure rate appeared to decrease with longer follow-up. However, important clinical outcomes, including persistent disease, recurrent hyperparathyroidism, repeat ablation procedures, and delayed parathyroidectomy, as well as relevant clinical variables associated with treatment failure, were inconsistently reported across the included studies. Consequently, formal analyses of potential risk factors for delayed recurrence or treatment failure were not feasible. Further large-scale multicenter randomized trials with standardized ultrasound guidance protocols and prolonged observation are needed to verify the long-term efficacy and safety of percutaneous MWA for PHP Finally, although the modified NOS was prespecified in our PROSPERO-registered protocol and consistently applied across all included studies, it may not comprehensively capture all sources of bias specific to single-arm observational studies. Therefore, the pooled findings should be interpreted with appropriate caution.

## 5 Conclusions

In conclusion, the available observational evidence suggests that percutaneous MWA under multiparametric ultrasound guidance may represent a promising minimally invasive option for appropriately selected PHPT patients. Although the available observational evidence suggests biochemical improvement and an acceptable safety profile, these findings should be interpreted with appropriate caution. Larger prospective comparative studies with standardized imaging protocols and longer follow-up are needed to confirm the efficacy and safety of this approach.

Future research should prioritize: (1) validation of the 1–2 mm non-enhancing rim on CEUS as a reliable criterion for confirming complete ablation; (2) multicenter prospective registries with unified protocols for patient selection, ablation parameters and follow-up schedules; (3) systematic documentation of clinical outcomes among patients without long-term cure, including recurrent hypercalcemia, repeat ablation and delayed surgery; and (4) external validation in other populations. Filling these research gaps will help to standardize ultrasound guidance protocols for percutaneous MWA and clarify its indications, contraindications and expected clinical outcomes. .

## Data Availability

The original contributions presented in the study are included in the article/**Supplementary Material**. Further inquiries can be directed to the corresponding author.

## Supplementary material

Supplementary Figure 1 Leave-one-out sensitivity analyses. **(A)** 6-month clinical cure rate; **(B)** End of study clinical cure rate; **(C)** Patient-level overall complication rate; **(D)** 12-month VRR. No single study disproportionately influenced the pooled effect estimates, confirming the statistical stability of the primary outcomes.
