# Supplementary material for: Percutaneous microwave ablation of hyperfunctioning parathyroid lesions under multiparametric ultrasound guidance for primary hyperparathyroidism: a systematic review and meta-analysis
Source: Front Oncol. 2026 Sep 21;16:1921204. doi: 10.3389/fonc.2026.1921204 (PMC13634870; doi:10.3389/fonc.2026.1921204)
Supplement: Supplementary file 1 [file Table1.docx]

**Supplementary Table S1. Detailed search strategies for each database**

| **Database** | **Search Strategy** |
| --- | --- |
| PubMed | ("Microwave Ablation"[MH] OR "microwave ablation" OR "MWA") AND ("Ultrasonography"[MH] OR ultrasound OR "ultrasound-guided" OR "ultrasound guidance" OR "multiparametric ultrasound" OR "MPUS" OR "CEUS" OR "contrast-enhanced ultrasound" OR elastography OR "shear wave elastography" OR "SWE" OR "strain elastography" OR Doppler OR "color Doppler" OR "CDUS") AND ("Hyperparathyroidism"[MH] OR "Hyperparathyroidism, Primary"[MH] OR parathyroid OR "parathyroid adenoma" OR hyperparathyroidism OR "primary hyperparathyroidism" OR "PHPT") |
| Cochrane Library | ("microwave ablation" OR "MWA") AND (ultrasound OR "ultrasound-guided" OR "ultrasound guidance" OR "multiparametric ultrasound" OR "MPUS" OR "CEUS" OR "contrast-enhanced ultrasound" OR elastography OR "shear wave elastography" OR "SWE" OR "strain elastography" OR "Doppler" OR "color Doppler" OR "CDUS") AND (parathyroid OR "parathyroid adenoma" OR hyperparathyroidism OR "primary hyperparathyroidism" OR "PHPT") |
| Scopus | TITLE-ABS-KEY ("microwave ablation" OR "MWA") AND TITLE-ABS-KEY (ultrasound OR "ultrasound-guided" OR "ultrasound guidance" OR "multiparametric ultrasound" OR "MPUS" OR "CEUS" OR "contrast-enhanced ultrasound" OR elastography OR "shear wave elastography" OR "SWE" OR "strain elastography" OR "Doppler" OR "color Doppler" OR "CDUS") AND TITLE-ABS-KEY (parathyroid OR "parathyroid adenoma" OR hyperparathyroidism OR "primary hyperparathyroidism" OR "PHPT") |
| EBSCOhost (Academic Search Ultimate) | ("microwave ablation" OR "MWA") AND (ultrasound OR "ultrasound-guided" OR "ultrasound guidance" OR "multiparametric ultrasound" OR "MPUS" OR "CEUS" OR "contrast-enhanced ultrasound" OR elastography OR "shear wave elastography" OR "SWE" OR "strain elastography" OR "Doppler" OR "color Doppler" OR "CDUS") AND (parathyroid OR "parathyroid adenoma" OR hyperparathyroidism OR "primary hyperparathyroidism" OR "PHPT") |
| Web of Science Core Collection | TS=("microwave ablation" OR "MWA") AND TS=("ultrasound" OR "ultrasound-guided" OR "ultrasound guidance" OR "multiparametric ultrasound" OR "MPUS" OR "CEUS" OR "contrast-enhanced ultrasound" OR elastography OR "shear wave elastography" OR "SWE" OR "strain elastography" OR "Doppler" OR "color Doppler" OR "CDUS") AND TS=(parathyroid OR "parathyroid adenoma" OR hyperparathyroidism OR "primary hyperparathyroidism" OR "PHPT") |

**Supplementary Table S2. Data extraction strategy for overlapping patient cohorts included in the meta-analysis**

| **Overlapping cohort** | **Baseline characteristics** | **Technical parameters** | **Biochemical outcomes** | **Overall complication analysis** | **Individual adverse events** | **Long-term follow-up** | **Denominator used** |
| --- | --- | --- | --- | --- | --- | --- | --- |
| Liu et al. (2022a, n = 91) + Liu et al. (2022b, n = 75) | Liu et al., 2022a [33] | Liu et al., 2022a [33] | Liu et al., 2022b [34] | **Not eligible for pooled analysis** | **Liu et al., 2022b [34]** | Liu et al., 2022b [34] | Baseline characteristics and technical parameters: n = 91; biochemical outcomes, individual adverse events, and long-term follow-up: n = 75. No Liu dataset was included in the pooled overall complication analysis because a patient-level numerator meeting the predefined complication criteria could not be derived. |
| Wei et al. (2020, n = 67) + Wei et al. (2022, PSM, n = 87) | Wei et al., 2022 [35] | Wei et al., 2020 [36] | Wei et al., 2020 [36] | Wei et al., 2022 [35] | Wei et al., 2022 [35] | Wei et al., 2020 [36] | Baseline characteristics, overall complications, and individual adverse events: n = 87; technical parameters, biochemical outcomes, and long-term follow-up: n = 67. |

For each outcome, data were extracted from the publication providing the most complete and relevant information, while ensuring that each overlapping patient cohort contributed only once to each pooled analysis. Overall complication data were included only when the number of patients experiencing at least one predefined major or minor complication could be identified. Studies that reported only individual adverse-event counts without an identifiable patient-level numerator were retained for the descriptive adverse-event synthesis but were not eligible for pooled overall complication analysis.

**Supplementary Table S3. Sensitivity analyses of major pooled outcomes using restricted maximum likelihood estimation, Hartung–Knapp confidence intervals, and 95% prediction intervals.**

| **Outcome** | **Primary analysis (DL)** | **REML + HK estimate (95% CI)** | **95% prediction interval** | **τ²** | **I² (%)** | **Interpretation** |
| --- | --- | --- | --- | --- | --- | --- |
| **6-month clinical cure** | **86.80%** | **86.80% (10.46–99.73)** | **0.98–99.98** | **1.539** | **67.8** | **Consistent with the primary analysis** |
| **12-month clinical cure** | **82.37%** | **82.37% (77.19–86.57)** | **68.46–90.95** | **0** | **0** | **Consistent with the primary analysis** |
| **End-of-study clinical cure** | **75.13%** | **75.13% (33.50–94.77)** | **4.81–99.45** | **1.759** | **86.9** | **Consistent with the primary analysis** |
| **Overall complication** | **11.50%** | **11.41% (3.00–34.93)** | **0.84–66.13** | **0.677** | **50.3** | **Consistent with the primary analysis** |
| **12-month VRR** | **90.86%** | **90.86% (79.09–102.63)** | **64.94–116.78** | **52.453** | **1** | **Consistent with the primary analysis** |

**Abbreviations: CI, confidence interval; DL, DerSimonian–Laird; HK, Hartung–Knapp; PI, prediction interval; REML, restricted maximum likelihood; VRR, volume reduction rate.**

**Note: REML/HK sensitivity analyses yielded point estimates consistent with the primary DL analyses across all major pooled outcomes. Wider confidence and prediction intervals for some outcomes reflect greater statistical uncertainty associated with high heterogeneity and limited study numbers.**

| **Study** | **N** | **Permanent RLN injury** | **Transient hoarseness** | **Transient hypocalcemia** | **Transient hypoparathyroidism** | **Hematoma** | **Other transient procedure-related events** | **Patient-level overall complication** | **Included in pooled analysis** |
| --- | --- | --- | --- | --- | --- | --- | --- | --- | --- |
| **Liu 2016** | **15** | **0** | **1** | **0** | **0** | **0** | **None** | **1/15** | **Yes** |
| **Fan 2019** | **22** | **0** | **0** | **1** | **0** | **0** | **Immediate transient voice change (8); minor puncture-site bleeding (2)** | **1/22** | **Yes** |
| **Wu 2020** | **20** | **0** | **6** | **0** | **0** | **0** | **Procedure-related pain (12)** | **6/20** | **Yes** |
| **Guan 2026** | **25** | **0** | **0** | **0** | **0** | **0** | **Puncture-site pain (2)** | **0/25** | **Yes** |
| **Zhou 2022** | **40** | **0** | **6** | **4** | **1** | **0** | **Neck swelling (40); procedure-related pain (18); cough (2)** | **NR** | **No** |
| **Liu 2022** | **75** | **0** | **4** | **13** | **NR** | **0** | **Fever (2); procedure-related pain‡** | **NR** | **No** |
| **Wei 2022** | **87** | **2** | **4** | **4** | **29** | **0** | **None reported** | **NR** | **No** |
| **Zhang 2022** | **35** | **0** | **3** | **2** | **0** | **0** | **Foreign-body sensation/swallowing discomfort (5); procedure-related pain (4)** | **NR** | **No** |
| **Ni 2021** | **20** | **0** | **3** | **0** | **0** | **0** | **Transient numbness/choking with normal serum calcium (1)** | **3/20** | **Yes** |

**Supplementary Table S4. Detailed classification of adverse events reported in the included studies**

**Abbreviations: RLN, recurrent laryngeal nerve; N, total number of patients; NR, not reconstructable.**

**Supplementary Table S5. Last available follow‑up serum calcium and iPTH levels**

| **Study** | **Follow‑up (months)** | **Calcium (mmol/L)** | **iPTH (pg/mL)** |
| --- | --- | --- | --- |
| Liu/2016 | 32.8 ± 17.9 | 2.32 ± 0.12 | 54.5 ± 24.1 |
| Wu/2020 | 24 | 2.37 ± 0.11 | 38.6 ± 7.5 |
| Guan/2026 | 7.48 ± 4.8 | 2.51 ± 0.19 | 89.0 ± 54.3 |
| Liu/2022* | 30 | 2.38 ± 0.05 | 55.9 ± 17.1 |
| Wei/2020 | 24 | 2.38 ± 0.17 | 45.1 (22.8–120.4) † |
|  |  |  |  |
| *Data from the prospective multicenter cohort (Liu et al. Eur Radiol 2022).  †Median (range). Note: Preoperative values are provided in Table 2 of the main text. Studies without long‑term follow‑up beyond 12 months (Fan/2019, Zhou/2022, Zhang/2022, Ni/2021) are not included here. | | | |
